# A comparative study of preclinical and clinical molecular imaging response to EGFR inhibition using osimertinib in glioblastoma

**DOI:** 10.1093/noajnl/vdaf022

**Published:** 2025-02-19

**Authors:** Benjamin M Ellingson, Quincy Okobi, Robert Chong, Rhea Plawat, Eva Zhao, Andrei Gafita, Ida Sonni, Saewon Chun, Emese Filka, Jingwen Yao, Donatello Telesca, Shanpeng Li, Gang Li, Albert Lai, Phioanh Nghiemphu, Johannes Czernin, David A Nathanson, Timothy F Cloughesy

**Affiliations:** Jonsson Comprehensive Cancer Center, University of California, Los Angeles, Los Angeles, California, USA; UCLA Brain Tumor Imaging Laboratory (BTIL), Department of Radiological Sciences, David Geffen School of Medicine, University of California Los Angeles, Los Angeles, California, USA; Department of Molecular & Medical Pharmacology, University of California, Los Angeles, Los Angeles, California, USA; Department of Neurology, University of California, Los Angeles, Los Angeles, California, USA; Department of Molecular & Medical Pharmacology, University of California, Los Angeles, Los Angeles, California, USA; Department of Molecular & Medical Pharmacology, University of California, Los Angeles, Los Angeles, California, USA; Jonsson Comprehensive Cancer Center, University of California, Los Angeles, Los Angeles, California, USA; Department of Molecular & Medical Pharmacology, University of California, Los Angeles, Los Angeles, California, USA; Jonsson Comprehensive Cancer Center, University of California, Los Angeles, Los Angeles, California, USA; Department of Molecular & Medical Pharmacology, University of California, Los Angeles, Los Angeles, California, USA; Department of Neurology, University of California, Los Angeles, Los Angeles, California, USA; Department of Neurology, University of California, Los Angeles, Los Angeles, California, USA; UCLA Brain Tumor Imaging Laboratory (BTIL), Department of Radiological Sciences, David Geffen School of Medicine, University of California Los Angeles, Los Angeles, California, USA; Department of Biostatistics, University of California, Los Angeles, Los Angeles, California, USA; Department of Biostatistics, University of California, Los Angeles, Los Angeles, California, USA; Department of Biostatistics, University of California, Los Angeles, Los Angeles, California, USA; Department of Neurology, University of California, Los Angeles, Los Angeles, California, USA; Department of Neurology, University of California, Los Angeles, Los Angeles, California, USA; Jonsson Comprehensive Cancer Center, University of California, Los Angeles, Los Angeles, California, USA; Department of Molecular & Medical Pharmacology, University of California, Los Angeles, Los Angeles, California, USA; Jonsson Comprehensive Cancer Center, University of California, Los Angeles, Los Angeles, California, USA; Department of Molecular & Medical Pharmacology, University of California, Los Angeles, Los Angeles, California, USA; Jonsson Comprehensive Cancer Center, University of California, Los Angeles, Los Angeles, California, USA; Department of Neurology, University of California, Los Angeles, Los Angeles, California, USA; Department of Molecular & Medical Pharmacology, University of California, Los Angeles, Los Angeles, California, USA

**Keywords:** EGFR, FDG PET, glioblastoma, imaging biomarker, osimertinib, molecular imaging

## Abstract

**Background:**

To demonstrate the potential value of ^18^F-fluorodeoxyglucose positron emission tomography (^18^F-FDG PET) as a rapid, non-invasive metabolic imaging surrogate for pharmacological modulation of EGFR signaling in EGFR-driven GBM, we synchronously conducted a preclinical imaging study using patient-derived orthotopic xenograft (PDOX) models and validated it in a phase II molecular imaging study in recurrent GBM (rGBM) patients using osimertinib.

**Methods:**

A GBM PDOX mouse model study was performed concurrently with an open-label, single-arm, single-center, phase II study of osimertinib (NCT03732352) that enrolled 12 patients with rGBM with EGFR alterations. Patients received osimertinib daily and 3 ^18^F-FDG PET scans: two 24 h apart prior to dosing, and one 48 h after dosing.

**Results:**

GBM PDOX models suggest osimertinib has limited impact on both ^18^F-FDG uptake (+ 9.8%–+25.9%) and survival (+ 15.5%; *P* = .01), which may be explained by insufficient exposure in the brain (Kp_uu_: 0.30) required to robustly inhibit the EGFR alterations found in GBM. Treatment with osimertinib had subtle, but measurable decreases in the linear rate of change of ^18^F-FDG nSUV growth rate averaging −4.5% per day (*P* = .01) and change in ^18^F-FDG uptake was correlated with change in tumor growth rate (R^2^ = 0.4719, *P = *.0195). No metabolic (PERCIST) or radiographic (RANO) responses were seen, and no improvements in PFS or OS were observed.

**Conclusions:**

This study demonstrated the feasibility of using FDG PET as a clinically reliable imaging biomarker for assessing EGFR inhibition in GBM, while revealing osimertinib’s limited impact on both metabolic activity and tumor growth in GBM, findings that were concordant between preclinical and clinical observations.

**Key Points**

- ^18^F-FDG PET in patients exhibited a high degree of reproducibility between scans.
- Osimertinib decreased the linear rate of change of ^18^F-FDG nSUV growth rate.
- Translational studies in GBM PDOX confirmed osimertinib’s limited impact.

**Importance of the Study**

This concurrent preclinical and clinical phase II study using ^18^F-FDG PET and a modestly brain penetrant EGFR inhibitor, osimertinib, to evaluate glucose utilization, is the first to evaluate the metabolic effects of osimertinib in patients with EGFR activated recurrent GBM. Rapid evaluation of drug-induced changes in GBM metabolic activity was assessed by ^18^F-FDG PET concurrently in preclinical models and patients. Preclinical models suggested potential lack of efficacy attributed to the drug not having the ability to effectively target the unique EGFR alterations found in GBM, relative to those EGFR alterations found in NSCLC—its FDA approved indication. This lack of treatment effect was also observed in a small cohort of patients with EGFR-altered GBM, with a minimal impact on metabolic activity and subsequent tumor growth inhibition.

EGFR small molecule inhibition is effectively used in myriad of cancers, including first line treatment of EGFR mutant non-small cell lung cancer (NSCLC).^1^ EGFR amplification and/or mutations drive malignant transformation by increasing signaling flux through PI3K-AKT-mTOR and RAS-MAPK pathways, which in turn confer pro-tumorigenic properties such as cell proliferation, increased glucose metabolism, and apoptotic resistance.^2^ Given that EGFR is genetically altered in approximately 60% of glioblastoma (GBM) patients (amplification, extracellular domain mutation, rearrangement, and alternative splicing), considerable efforts have been directed toward repurposing EGFR TKIs approved for NSCLC for use in GBM.^3^

Despite the attractiveness of EGFR as a target in GBM, clinical trials using EGFR TKIs developed for non-CNS primary indications have yielded disappointing results in GBM trials thus far, with no FDA-approved medications. Both first-generation (e.g. erlotinib, gefitinib, lapatinib, and vandetanib) and second-generation (e.g. neratinib, afatinib, dacomitinib, and tesevatinib) EGFR TKIs have failed to demonstrate efficacy in GBM either as single agents or in combination with other therapies.^4–8^ Prior studies have demonstrated that combined EGFRvIII expression and retained PTEN predicted response to first-generation EGFR inhibitors erlotinib and gefitinib in GBM patients.^9^ These findings highlight the importance of molecular stratification in predicting treatment response, though subsequent trials with various EGFR inhibitors have shown limited efficacy even in molecularly selected populations. Importantly, GBM poses unique challenges to effective small molecule targeting, with prior failures attributed to multifactorial etiologies including insufficient blood–brain barrier (BBB) penetration, intratumoral EGFR heterogeneity, and unique extracellular domain mutations that are distinct in location and function from those observed in other cancers.^10–12^

Effective therapeutic response in EGFR altered GBM is achieved only when tumor cells receive sufficient drug exposures, which in turn lead to biologically relevant inhibition of downstream signaling.^13^ In support of this, initial negative studies with erlotinib identified a subset of GBM patients under oral treatment who underwent repeat resection; these patients demonstrated tissue-to-plasma ratios of 6–8%, which were deemed inadequate for intratumoral penetration.^14^ A subsequent phase II surgical trial found that oral gefitinib could concentrate in the brain tissue and dephosphorylate EGFR but had a limited effect on downstream signal transduction.^15^ Although phosphorylation of EGFR was decreased in the specific samples taken, spatial heterogeneity of drug exposure in brain tumors has been well documented to be highly variable, highlighting the difficulties in accurately evaluating tumors where repeat biopsies are not possible.^16^

Together, these studies provide alternative mechanistic explanations for poor clinical outcomes using EGFR TKIs; they also highlight the challenging nature of harvesting CNS tissue, which necessitates invasive procedures such as repeat craniotomy/biopsy. By contrast, traditional measures, such as radiographic response (eg, RANO criteria) and overall survival (OS)/progression-free survival (PFS), are non-invasive, yet are unable to provide more granular metabolic or molecular information. Clearly, there is an unmet need to develop and implement tools to understand dose response relationships for GBM EGFR small molecule inhibitor candidates.

^18^F-fluorodeoxyglucose positron emission tomography (^18^F-FDG PET) is ubiquitously used in a variety of cancer types to non-invasively quantify glucose uptake resulting primarily from elevated glycolysis and to measure clinical activity of an agent. Glycolysis is critical for GBM growth, proliferation, and survival^17,18^ and amplification and/or mutation in EGFR is known to drive heighted GBM glycolytic flux via activation of PI3K-AKT-mTOR and/or RAS-MAPK signaling pathways.^19,20^ Accordingly, successful ablation of aberrant EGFR signaling rapidly (hours) reduces glycolysis in GBM.^21^ This connection between EGFR signaling and glycolysis in GBM, together with clinical data in EGFR mutant NSCLC, suggests ^18^F-FDG PET might be a suitable as an early non-invasive pharmacodynamic biomarker for biologically meaningful inhibition of EGFR signaling in GBM^22–24^*before* changes in tumor growth arrest and induction of apoptosis.

Relative to other first-generation EGFR TKIs like erlotinib or gefitinib, osimertinib is a brain-penetrant (mouse Kp_uu_: 0.21–0.39^25–27^), third generation EGFR TKI that covalently binds to EGFR,^28^ with front line applications in NSCLC patients harboring either L858R or exon 19 deletion.^29–31^ Given osimertinib’s proven effectiveness in metastatic NSCLC, strong CNS activity in both lung cancer patients and lung cancer pre-clinical models,^27^ and pharmacokinetic studies establishing reasonable distribution throughout the brain,^32,33^ we posited osimertinib may have potential for a clinically observable and quantifiable metabolic imaging response in treating EGFR-altered GBM. To test this hypothesis, we synchronously conducted a preclinical imaging study to investigate target inhibition, ^18^F-FDG uptake, and survival in patient-derived orthotopic EGFRvIII mutant GBM preclinical models as well as a phase II clinical study of osimertinib in recurrent GBM patients harboring EGFR amplification or mutation, and quantified the metabolic and radiographic changes.

## Materials and Methods

### Preclinical Methods

#### GBM cell culture conditions.—

The GBM39 cell line was a gift from David James. Primary GBM cells (GBM39) were established and maintained in gliomasphere conditions consisting of DMEM/F12 (Gibco), B27 (Invitrogen), penicillin–streptomycin (Invitrogen), and GlutaMAX (Invitrogen) supplemented with heparin (5 μg/mL, Sigma), EGF (20 ng/mL, Sigma), and FGF (20 ng/mL, Sigma). All cells were grown under 37°C, 20% O_2_, and 5% CO_2_ and were routinely monitored and tested negative for the presence of mycoplasma with a commercially available kit (MycoAlert, Lonza). At the time of experiments, the GBM39 line was used at fewer than fifteen passages. All cells were authenticated by short tandem repeat analysis.

#### Non-GBM cell culture conditions.—

Non-small cell lung cancer cell line PC9 was gifted from Dr. Peter Clark and was cultured in RPMI 1640 with 2 mM Glutamine (Gibco) and supplemented with 10% fetal bovine serum. Cells were dissociated to single cell suspensions with TrypLE (Thermofisher) and resuspended in its respective media. All cells were grown under 37°C, 20% O_2_, and 5% CO_2_ and were routinely monitored and tested negative for the presence of mycoplasma with a commercially available kit (MycoAlert, Lonza). At the time of experiments, the GBM39 line was used at fewer than ten passages. Characteristically, PC9 cells harbor an EGFR exon 19 deletion and are highly sensitive to EGFR TKI.

#### Mice.—

Female NOD *scid* gamma (NSG) mice, 6–8 weeks of age, were purchased from the University of California Los Angeles (UCLA) Medical Center animal-breeding facility and Jackson Laboratories. All mice were kept under defined pathogen-free conditions at the AAALAC-approved animal facility of the Division of Laboratory Animals at UCLA. Sample sizes were chosen based on estimates from pilot experiments. Mice were euthanized when moribund or reached a 25% loss in body weight. Investigators were not blinded to group allocation or assessment of outcome. All studies using mice were in accordance with UCLA OARO protocol guidelines and in accordance with UCLA Animal Research Committee protocol guidelines. All experiments using mice were approved by the Institutional Animal Care and Use Committees at UCLA.

#### Gliomasphere-derived orthotopic xenografts.—

To produce gliomasphere-derived orthotopic xenograft tumors, gliomaspheres were transduced with the Gaussia-luciferase reporter^34^ to enable non-invasive quantification of tumor burden as well as endpoint GFP-guided microdissection of the tumor tissue from the surrounding normal brain. Gliomaspheres were dissociated and injected (2.5 × 10⁵ cells per injection) into the right striatum of the brain in female NSG mice (7–9 weeks old). Injection coordinates were 2 mm lateral and 1 mm posterior to bregma, at a depth of 2 mm. Tumor burden was monitored based on secreted Gaussia luciferase, and after a 10-fold increase in RLU from baseline, mice were randomized into treatment arms. Tumor burden was assessed twice per week for the duration of the study.

#### Secreted Gaussia luciferase measurements.—

Cells were infected with a lentiviral vector containing a secreted Gaussia luciferase-encoding reporter gene (Targeting Systems no. GL-GFP) and intracranially implanted as described into NSG mice. To measure the levels of secreted Gaussia luciferase, 6 μL of blood was collected from the tail vein and immediately mixed with 50 mM EDTA to prevent coagulation. Secreted Gaussia luciferase activity was obtained by measuring chemiluminescence after injection of 100 μL of 100 μM coelentarazine (Nanolight) in a 96-well plate, as described before.^34^

#### Intracranial mouse treatment studies.—

GBM39 or PC9 cells were intracranially injected into NSG mice as described above. When the tumors were engrafted and began an exponential growth phase by Gaussia luciferase measurement, mice were randomized into treatment arms and initiated treatment by oral gavage with either vehicle (2% hydroxypropylcellulose, pH 4.5) or osimertinib (10 mg/kg or 25 mg/kg). Mice were treated for 5 days followed by 2 days of no treatment each week until endpoints were reached. Mice were euthanized when moribund or reached a 25% loss in body weight. All studies were in accordance with UCLA Animal Research Committee protocol guidelines.

#### Intracranial delayed 18F-FDG PET/CT mouse imaging.—

 For baseline ^18^F-FDG PET scans, mice were prewarmed, anesthetized with 2% isoflurane, and intravenously injected with 70 μCi of ^18^F-FDG. After 1 h of unconscious uptake, the mice were taken off anesthesia but kept warm for another 5 h of uptake. Six hours after the initial administration of ^18^F-FDG, mice were imaged with a G8 PET/CT scanner (Sofie Biosciences). After imaging, all mice were dosed with osimertinib at 10 mg/kg or 25 mg/kg and 72 h later were subjected to the same imaging procedure. As described above, quantification was performed by drawing 3D regions of interest in AMIDE software, as previously described.^35^ The 24-h treatment time point was the earliest time point that fit within logistical constraints including the amount of time required for adequate probe decay for subsequent imaging, the ^18^F-FDG production schedule and the hours of operation of the imaging center.

#### Pharmacokinetic studies.—

Male CD-1 mice were treated by oral gavage with 10 mg/kg or 25 mg/kg of EGFR inhibitor, as performed previously (37). Mice were euthanized and whole blood and brain tissue were collected at 0, 0.5, 1, 2, 4, 7, and 24 h post treatment (*n* = 2 mice per time point). Whole blood from mice was centrifuged to isolate plasma. EGFR inhibitors were isolated by liquid–liquid extraction from plasma: 50 µL plasma was added to 150 µL acetonitrile and 5 pmol gefitinib internal standard. Mouse brain tissue was washed with 2 mL cold PBS and homogenized using a tissue homogenizer in 2 mL cold water. EGFR inhibitors were then isolated and reconstituted in a similar manner by liquid–liquid extraction: 100 µL brain homogenate was added to 5 pmol gefitinib internal standard and 300 µL acetonitrile. After vortex mixing, the samples were centrifuged. The supernatant was removed and evaporated by a rotary evaporator and reconstituted in 100 µL 50:50:0.1 water:acetonitrile:formic acid.

#### Protein binding.—

Protein binding was performed using 8K MWCO rapid equilibrium dialysis plates (Thermofisher). Briefly, CD-1 mouse plasma or homogenized brain tissue was mixed to a final concentration of 5 µM EGFR inhibitor and added to the sample side of the dialysis plate with PBS as the dialysate. Plates were dialysed at 37°C on a shaker for 6 h followed by the extraction methods mentioned above.

#### EGFR inhibitor detection.—

Chromatographic separations were performed on a 100 × 2.1 mm Phenomenex Kinetex C18 column (Kinetex) using the 1290 Infinity LC system (Agilent). The mobile phase was composed of solvent A: 0.1% formic acid in Milli-Q water, and B: 0.1% formic acid in acetonitrile. Analytes were eluted with a gradient of 5% B (0–4 min), 5–99% B (4–15 min), 99% B (15–20 min), and then returned to 5% B for 10 min to re-equilibrate between injections. Injections of 20 µL into the chromatographic system were used with a solvent flow rate of 0.10 mL/min. Mass spectrometry was performed on the 6460 triple quadrupole LC/MS system (Agilent). Ionization was achieved by using electrospray in the positive mode and data acquisition was made in multiple reactions monitoring mode. Analyte signal was normalized to the internal standard and concentrations were determined by extrapolating on to the calibration curve (10, 100, 1000, 4000 nM). EGFR inhibitor brain concentrations were adjusted by 1.4% of the mouse brain weight for the residual blood in the brain vasculature as described previously.^36^

#### Reagents and antibodies.—

The chemical inhibitor osimertinib (Selleck Chemicals) was dissolved in DMSO for all in vitro studies. For in vivo studies, stock solutions of 10 mg/kg and 25 mg/kg in sterile 2% hydroxypropylcellulose (Ashland), pH 4.5 were stored at −20°C until use. Mice were treated at 10 mL/kg body weight. The following antibodies for immunoblotting were obtained from the listed sources: p-EGFR Y1086 (Thermofisher, 36-9700), t-EGFR (Millipore, 06-847) p-AKT S473 (Cell Signaling, 4060), t-AKT (Cell Signaling, 4685), p-ERK T202/Y204 (Cell Signaling, 4370), t-ERK (Cell Signaling, 4695), p-S6 S235/236 (Cell Signaling, 4858), t-S6 (Cell Signaling, 2217), and β-Actin (Cell Signaling, 3700).

#### Immunoblotting.—

Cells were collected and lysed in RIPA buffer (Boston BioProducts) containing Halt™ Protease and Phosphatase Inhibitor (Thermofisher). Lysates were centrifuged at 14,000 g for 15 min at 4°C. Protein samples were then boiled in NuPAGE LDS Sample Buffer (Thermofisher) and NuPAGE Sample Reducing Agent (Thermofisher), separated using SDS-PAGE on 12% Bis-Tris gels (Thermofisher), and transferred to nitrocellulose membranes (GE Healthcare). Immunoblotting was performed per antibody’s manufacturer’s specifications. Membranes were developed using the SuperSignal™ system (Thermofisher) and imaged using the Odyssey Fc Imaging System (LI-COR). Signal quantification was performed using the Image Studio™ software (LI-COR).

#### Ex vivo immunoblot studies.—

GBM39 or PC9 cells were intracranially injected as described into NSG mice. When the tumors were engrafted and began an exponential growth phase by Gaussia luciferase measurement as described above, mice were randomized into treatments arms and were treated with either vehicle or osimertinib (10 mg/kg or 25 mg/kg) for 3 consecutive days. Mice were then euthanized, and tumors were isolated by macro dissection by GFP fluorescence. Tumors were lysed by sonication in RIPA buffer (Boston BioProducts) containing Halt™ Protease and Phosphatase Inhibitor (Thermofisher). The immunoblotting protocol above was then performed on lysates.

### Clinical Trial Methods

#### Study design.—

This investigator-initiated trial was an open-label, single-arm, single-center, phase II study of osimertinib in patients with recurrent glioblastoma after failure of initial therapy that harbored EGFR alternations and were p53 wild-type (ClinicalTrials.gov #NCT03732352). The study was sponsored by UCLA with support from AstraZeneca, who provided Osimertinib and funds to conduct the study through a research grant. The study was conducted under IND #142439. The primary objectives were to investigate the test–retest variance of ^18^F-FDG PET and to determine whether osimertinib would cause a significant decrease in glucose utilization as determined using early, post-treatment FDG PET using a pre-planned sample size of 12 patients. The phase II trial was designed as a descriptive study to evaluate early metabolic changes using FDG-PET and was not powered for efficacy endpoints. A sample size of 12 patients was selected to provide preliminary evidence of metabolic response while minimizing patient exposure to an unproven therapy. Other objectives included efficacy, safety, and tolerability. The study was implemented and reported in accordance with the Good Clinical Practice Guidelines, with applicable local regulations, and the Declaration of Helsinki. The study protocol was approved by the ethics committee at UCLA. Patients were required to provide written informed consent.

#### Patients.—

Male or female patients aged 18 years or older and Karnofsy performance status ≥ 60 with recurrent supratentorial glioblastoma harboring any EGFR abnormality (including amplification and/or mutation) and p53 Wild-type as determined by local or central Clinical Laboratory Improvement Amendments–accredited laboratories before study entry were eligible. The p53 wild-type requirement was included based on preclinical data suggesting enhanced sensitivity to EGFR inhibition in this molecular context.^22^ Evidence of recurrence or progression for study entry was done through the evaluation of pre-progression and progression imaging. All patients or their legally authorized representatives provided written informed consent prior to enrollment. All patients were required to have contrast enhancing target lesions that were greater than or equal to 1 × 1 × 1 cm^3^. Other typical criteria were used for ability to take oral medications, recovery from prior therapies and toxicities, limited laboratory abnormalities, life expectancy.

#### Treatment.—

Patients were intended to receive oral osimertinib 240 mg daily for 3 days and then 160 mg daily in 28-day cycles until disease progression or unacceptable toxicity. Criteria for dose adjustment for toxicity or tolerability issues were predefined.

#### Safety.—

Safety evaluation took place at each cycle and ad hoc. Clinical chemistry, hematology, coagulation, and serum pregnancy were obtained. Cardiac toxicity was evaluated through electrocardiogram and echocardiogram. Adverse events were characterized using NCI CTCAE, Version 4.03 and slit lamp exams were performed to evaluate ophthalmic toxicity.

#### Clinical outcomes.—

The clinical component of this study was not designed to provide a statistical evaluation of clinical outcomes, such as responses, progression free survival, or survival. Results of these endpoints are descriptive only. Response was defined using the modified RANO criteria^37^ (comparable to RANO 2.0 with confirmation of progression^38^) and PFS was defined as the time from randomization to disease progression by RANO or death from any cause. Survival was defined from the day of randomization to death. Any patient lost to follow up or still alive at the time of evaluation censored.

#### Magnetic resonance imaging (MRI).—

All subjects had MR images acquired at all pre-treatment and post-treatment follow-up visits according to the international standardized brain tumor imaging protocol,^39^ including T2 weighted, T2-weighted FLAIR, diffusion-weighted images, and parameter matched, 1–1.5 mm isotropic 3D T1-weighted scans before and following injection of gadolinium-based contrast agents. Radiographic assessment by MRI for tumor staging by modified RANO^37^ was performed using historic scans spanning 6 months prior to enrollment and a scan at screening (within 14 days of enrollment) to confirm the presence of growing contrast enhancing tumor and measurable baseline size for eligibility. Additionally, a “hyperacute” pre-treatment baseline scan at the time of the second pre-treatment ^18^F-FDG PET scan, within one day of the start of osimertinib treatment. Subsequent MRI scans were performed every 8 ± 1 weeks starting from cycle 3 day 1 per patients’ standard of care until disease progression via modified RANO.^37^ Brain imaging data were collected during long-term follow-up if the subjects were off treatment for any reason other than disease progression.

#### 18F-FDG PET imaging.—

Three ^18^F-FDG PET scans were performed: 2 scans prior to osimertinib dosing, 18 to 54 h apart, and one scan 24 to 72 h after the initial dose of 240 mg osimertinib. Significant changes in recurrent glioblastoma growth can occur within a week.^40^ Therefore, to isolate early pharmacodynamic changes from changes in tumor size that might confound interpretation, post-treatment ^18^F-FDG PET scans were acquired within 72 h of osimertinib. The Siemens Biograph 64 TrueV HI-REZ and the Siemens Biograph mCT combined PET/CT systems were used. Patients were instructed to fast for at least 4 h prior to injection. After an intravenous injection of ^18^F-FDG at 5 mCi and after an uptake period of 90 min, static ^18^F-FDG PET images were acquired for 15 min. To minimize a patient’s head motion, hypoallergenic medical tape was applied across the forehead and head cushion before the PET acquisition. PET emission data were corrected for photon attenuation, photon scatter, and random coincidences were reconstructed by use of iterative reconstruction with ordered-subset expectation maximization (16 iterations) and a gaussian filter with a full width at half maximum of 4 mm.

#### Determination of 18F-FDG PET region of interest.—

 All ^18^F-FDG PET images were linearly registered (6 °C of freedom) to pre-treatment, post-contrast T1-weighted MRI scans, resulting in fusion of the FDG PET and the MRI images (**Supplementary Figure S1A****–****C**). Using this alignment, contrast enhancing regions of interest were contoured on post-contrast T1-weighted images and directly applied to the respective FDG PET images for FDG uptake measurement (**Supplementary Figure S1D****–****E**). A background correction method was applied by referencing SUVs to the cerebellum to obtain the normalized standard uptake value.^41^

#### Research tissue collection.—

Archived tumor tissue, preferably from the most recent biopsy/resection, of 20 or more freshly cut, unstained FFPE slides (4–5 μm each) were obtained at screening for retrospective central laboratory confirmation of EGFR amplification/mutation status and p53 mutation status. Corresponding pathology report was also required. Patients were enrolled based on the documented EGFR/p53 status from the previous assays completed locally.

#### Statistical analyses.—

Unless otherwise specified, comparisons were made with 2-tailed unpaired Student’s *t*-tests, and *P* values < .05 were considered statistically significant. Data represent mean ± SD values unless otherwise indicated. Statistical analyses were calculated in Prism 9.0 (GraphPad) unless otherwise specified. The code for elastic net and cross validations was written in R (v4.1.2). For all in vitro and in vivo experiments, no statistical method was used to predetermine sample size, and no samples were excluded. For in vivo tumor measurements, 2-way ANOVAs were used for comparisons between groups. As described above, all mice were randomized before studies.

To investigate the relationship between GBM39 and PC9 survival and the biomarkers among the mice, we carried out regression analyses to explain the variance of GBM39 survival by each of the 2 biomarkers, including EGFR activity (%) and ^18^F-FDG uptake change (%), respectively. Our analyses consider both linear and log-linear models association. R-squared measures for non-linear models were obtained using previously described approaches.^42^

We modeled longitudinal mean ^18^FDG-PET SUV trajectories using a linear mixed effects regression model, accounting for the effect of osimertinib through a change in slope after the second baseline measurement. Our model included random intercepts and slopes before and after treatment, to account for subject-level effect heterogeneity and within-subject patterns of dependence. Longitudinal log tumor volume trajectories are modeled using a linear mixed effects regression model. Specifically, we account for the effect of osimertinib through a change in slope after treatment. Our model included random intercepts, to account for subject-level heterogeneity and within-subject patterns of dependence. In both longitudinal analyses, estimates of fixed (population-level) effects are obtained maximizing the model marginal likelihood, variance components estimates are based on the restricted maximum likelihood. Confidence intervals and standard errors associated with percent change estimates are obtained using a parametric bootstrap procedure.

## Results

### ^18^F-FDG PET as a Predictive Pharmacodynamic Marker of Response in Preclinical Models

**Figure 1.**
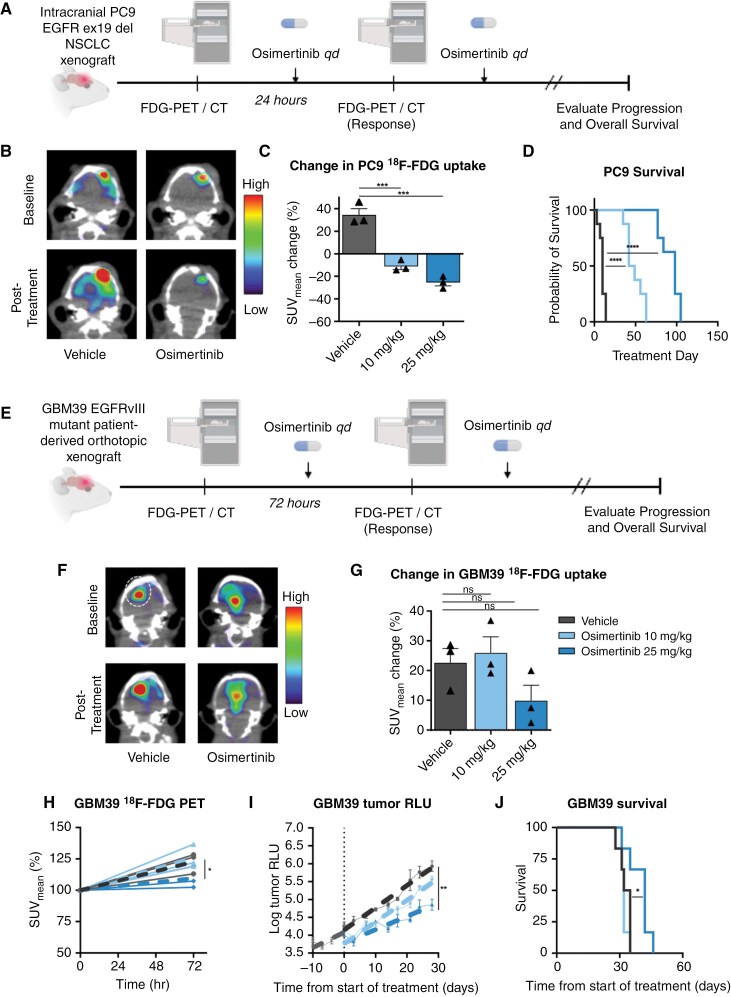
**Preclinical** ^**18**^ **F-FDG PET imaging and therapeutic response to osimertinib in NSCLC and GBM intracranial models.** (A) Preclinical intracranial NSCLC study schema. Mice are implanted with the NSCLC model. After engraftment, mice receive a baseline ^18^F-FDG PET scan followed by osimertinib treatment for 1 day. Another ^18^F-FDG PET scan is then performed. RLU measurements are taken twice weekly until death. (B) Example overlaid ^18^F-FDG PET/CT scans of NSCLC tumor-bearing mice treated with vehicle or osimertinib. (C) Change in ^18^F-FDG uptake after osimertinib treatment in the intracranial NSCLC mouse model. (D) Kaplan-Meier curves of overall survival (*n* = 8). Responses to both the 10 mg/kg and 25 mg/kg osimertinib treatment were significantly different from controls. (E) Preclinical intracranial GBM study schema. Mice are implanted with a GBM patient-derived orthotopic model. After engraftment, they receive a baseline ^18^F-FDG PET scan followed by daily osimertinib treatment for 3 days. Another ^18^F-FDG PET scan is then performed. RLU measurements are taken twice weekly until death. (F) Example overlaid ^18^F-FDG PET/CT scans of GBM tumor-bearing mice treated with vehicle or osimertinib. (G) Change in ^18^F-FDG uptake after osimertinib treatment in the intracranial GBM39 mouse model (*n* = 3). (H) Individual mouse nSUV_mean_ trajectories of vehicle and treatment response. Projected mean linear rate of changes are plotted (dotted lines) and a significant reduction in the rate of change of the 25 mg/kg osimertinib was observed. (I) Log of gaussia RLU over time before and after treatment. (J) Kaplan–Meier curves of overall survival (*n* = 6). Only the 10 mg/kg osimertinib treatment was not significantly different from the control treatment. Using a mixed effects regression model, mean linear growth rates are plotted (dotted lines) before and after start of treatment. *P < .05, **P < .01, ***P < .001, ****P < .0001.

Therapeutic benefit to EGFR TKI in NSCLC models and patients is linked to rapid changes in glycolysis with EGFR TKI.^23,43^ To determine whether osimertinib can reduce ^18^F-FDG uptake and drive tumor responses in an intracranial model of EGFR-mutant NSCLC, we implanted EGFR exon 19 deleted PC9 cells into the brains of NSG mice. Once exponential tumor growth was achieved, we subjected tumor-bearing mice to 2 ^18^F-FDG PET scans spaced 72 h apart (Figure 1A), before and after treatment with vehicle, 10 mg/kg, or 25 mg/kg osimertinib, with 25 mg/kg predicted to be within the range of clinically relevant doses based on matching free plasma exposure at steady state and taking into account free plasma exposure of the active metabolite, AZ5104.^28,44^ Both 10 mg/kg and 25 mg/kg osimertinib treatment resulted in substantial, dose-dependent decreases in tumor ^18^F-FDG uptake in the PC9 NSCLC intracranial xenografts (Figure 1B–C; 10 mg/kg: −11.1%, *P* < .001; 25 mg/kg: −25.4%, *P* < .001). Median survivals of 10 days, 45.5 days, and 98 days were observed with treatments of vehicle, osimertinib 10 mg/kg, and osimertinib 25 mg/kg, respectively (Figure 1D) and significant improvements in survival were observed with both doses of osimertinib (*P* < .001) compared to vehicle.

To better understand osimertinib activity in GBM, we developed an intracranial, patient-derived EGFRvIII mutant GBM xenograft model (GBM39) and again obtained ^18^F-FDG PET scans before and after osimertinib treatment (Figure 1E). GBM tumors imaged before and after vehicle treatment displayed an increase in mean tumor SUV (Figure 1F–G; 22.62% ± 6.8%), while osimertinib at both doses had at best a minimal effect on GBM39 ^18^F-FDG uptake (Figure 1G; 10 mg/kg: + 25.9% ± 2.5%; 25 mg/kg: + 9.8% ± 4.3%). Data suggest the linear change in^18^F-FDG uptake was only slightly, but significantly, altered at the 25 mg/kg dose (Figure 1H; *P <* .05), with virtually no effect observed at lower doses. Similarly, linear tumor growth rates evaluated using gaussia luciferase^34^ as a measure of tumor burden showed only a subtle, yet significant alteration in growth rate at a dose of 25 mg/kg compared with vehicle (Figure 1I; *P < *.05). No significant improvement in survival was observed with 10 mg/kg osimertinib (Figure 1J; median survival: 32 days; *P* = .354) compared to vehicle (median survival: 35 days) and a very modest, albeit significant, survival benefit was observed with 25 mg/kg osimertinib (median survival: 42 days, *P* = .041) (the maximum tolerated dose in NSG mice (**Supplementary Figure S2**). Thus, preclinical experiments suggest osimertinib was unable to significantly alter metabolic activity and growth properties in GBM, while intracranial EGFR-mutated NSCLC tumors showed both reduced ^18^F-FDG uptake and improved outcomes.

### Inhibition of EGFR Signaling by EGFR TKIs Is Correlated with Inhibition of ^18^F-FDG Uptake and Improved Survival

**Figure 2.**
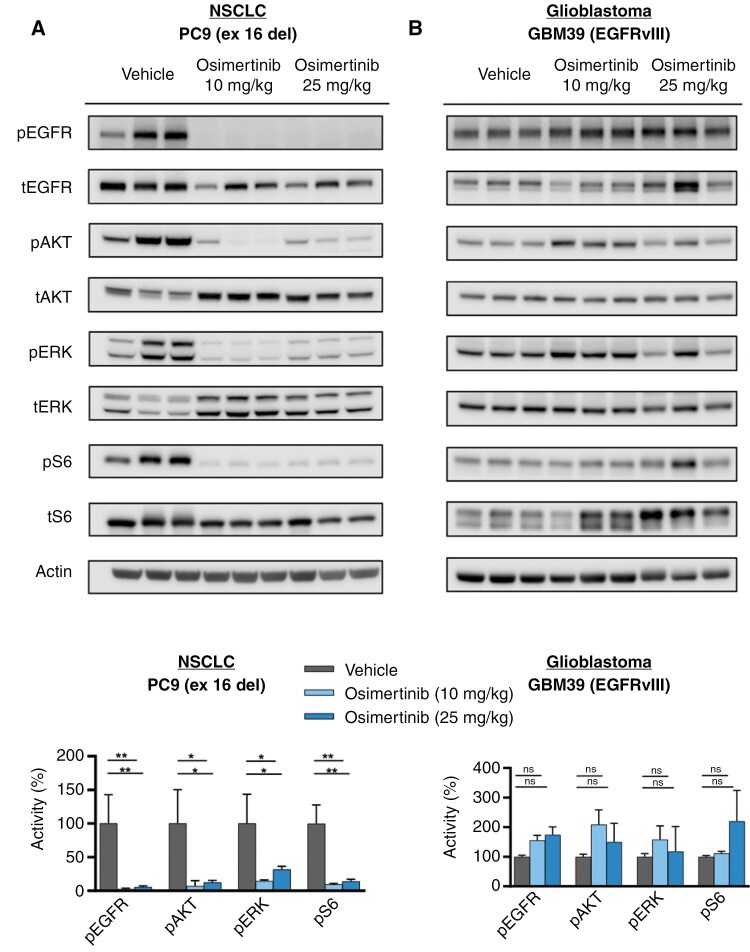
**In vivo inhibition of EGFR signaling by EGFR TKIs is correlated with inhibition of** ^**18**^ **F-FDG uptake and improved survival.** Immunoblots of phosphorylated and total EGFR, and downstream kinases AKT, ERK, and S6 from in vivo tumors (*n* = 3) for (A) PC9 (NSCLC) and (B) GBM39 (GBM) models. Quantification of kinase phosphorylation from (C) PC9 and (D) GBM39 tumors. Results show that osimertinib has limited inhibitory activity against in vivo intracranial GBM models but is effective in NSCLC intracranial models. Phosphorylated protein activity is normalized to total protein levels. *P < .05, **P < .01.

Rapid changes in glycolysis with EGFR TKI are coupled to robust inhibition of EGFR signaling.^22^ Thus, we asked whether the distinct FDG responses and outcomes with the osimertinib on the tumor xenografts could be explained by their unique pharmacodynamic properties on EGFR signaling. Intracranial NSCLC tumor tissues from vehicle or osimertinib treated mice were extracted and analyzed for activation of exon 19 deleted EGFR and downstream kinase activity (**Figure 2A**). These analyses demonstrated strong inhibition of both EGFR and downstream kinases for both doses of osimertinib (**Figure 2C**). With 10 mg/kg osimertinib, EGFR activation in intracranial PC9 tumors was inhibited by 97.5% (*P* = .007) and the downstream kinases pAKT, pERK, and pS6 were inhibited by 93.2%, 85.5%, and 90.2%, respectively (*P* = .019, *P* = .014, and *P* = .001). The higher osimertinib dose also elicited a strong degree of EGFR pathway inhibition, with EGFR inhibited by 94.5% (*P* = .008) and the downstream kinases pAKT, pERK, and pS6 inhibited by 87.7%, 68.3%, and 86.0%, respectively (*P* = 0.025, *P* = .037, and *P* = .001). In contrast to EGFR-mutated NSCLC models, EGFR signaling in GBM models failed to display reduction of both the activation of EGFRvIII and downstream kinases upon osimertinib treatment (**Figure 2B**). While the higher dose osimertinib treatment (25 mg/kg), EGFRvIII activation was not significantly inhibited. No significant changes in the activation of downstream kinases pAKT, pERK, and pS6 were observed (**Figure 2D**). These preclinical data suggest that changes in FDG uptake could serve as a surrogate marker for tumor pharmacodynamics in response to EGFR TKI therapy.

### Concurrent Clinical Trial Patient Characteristics

In parallel to the preclinical evaluations, twelve patients with recurrent GBM were enrolled in a single arm phase II clinical trial (NCT03732352). The median follow-up time was 164 days (range: 55–408 days). The baseline patient characteristics are summarized in **Table 1**. Ten of 12 patients were female, and the median age at enrollment was 57.5 years with a range of 44–79. Enrolled patients were on their first to third recurrence, with Karnofsky Performance status ranging from 70 to 90. Median baseline enhancing tumor volume was 48 mL, and non-enhancing volume was 142.5 mL, with an enhancing tumor growth rate of 23.65 mL/28 days. All patients exhibited confirmed radiographic progression by mRANO prior to treatment. Osimertinitb was administered orally at 240 mg a day for 3 days and then 160 mg daily until tumor progression, subject withdrawal of consent or unacceptable toxicity. As of the cutoff date February 18, 2021, 12 of 12 patients had discontinued osimertinib (one by withdrawal of consent and 11 by disease progression by investigator response determination). The 12 patients went on to receive 33 cycles of osimertinib. Dexamethasone was administered at the investigator’s discretion.

**Table 1.** Patient Characteristics.

Characteristic	Median [Range]
Age	57.5 [44–79]
Baseline KPS	85 [70–90]
Prior recurrences	1.5 [1–3]
Baseline steroid dose [mg/day]	2 [0–16]
Baseline enhancing volume [mL]	48 [10.7–92.9]
Baseline FLAIR volume [mL]	142.5 [48.1–219.6]
Confirmed radiographic PD prior to TX	12/12 (100%)
Baseline enhancing tumor growth rate [mL/28 days]	23.7 [4.0–77.1]
Immediate baseline ^18^F-FDG PET (tumor/cerebellum) [mean nSUV]	0.93 [0.73–1.40]

#### Safety.

Osimertinib was generally well tolerated, and the medication was not associated with any new or previously unreported toxicities (Supplementary Table S1). In total, 3 out of the 12 patients experienced Grade 3 treatment emergent adverse events, with mental status changes being the most common side effect observed in 4/12 patients (33%). Two patients exhibited cerebral edema as determined by MRI, 2 patients developed hyponatremia and the remaining grade 3 treatment emergent adverse events were only experienced by one patient each. No patients exhibited a grade 4 treatment emergent adverse event.

### Clinical ^18^F-FDG PET Double Baseline Demonstrates Minimal Variance and High Reliability

**Figure 3.**
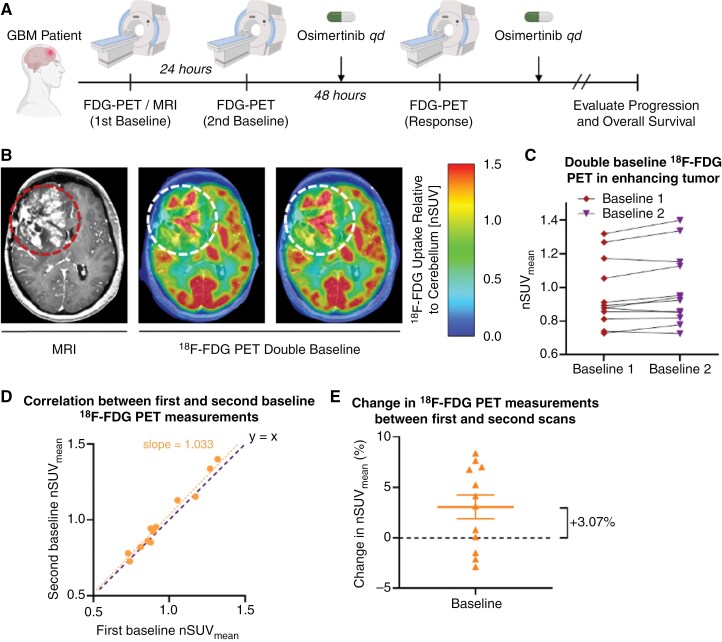
**Clinical trial schema and estimation of reproducibility for** ^**18**^ **F-FDG uptake in recurrent GBM patients.** (A) Clinical study schema. Patients receive two baseline ^18^F-FDG PET scans followed by 240 mg of daily osimertinib treatment for 3 doses in 48 h. A third ^18^F-FDG PET scan is then performed and 160 mg daily osimertinib treatment continues until tumor progression. (B) Example clinical baseline 1 and baseline 2 ^18^F-FDG PET scan demonstrating minimal variance between baseline scans. Dotted lines indicate regions of tumor (C) Individual first and second baseline ^18^F-FDG PET measurements of the enhancing tumor of 12 patients. (D) Strong correlations between the first and second baseline scans are observed with an overall increasing trend of nSUV_mean_. (E) Mixed-effects regression estimates of the percent change in nSUV_mean_ from baseline 1 to baseline 2.

To establish the variance in ^18^F-FDG PET uptake in GBM tumors without treatment, 2 pre-treatment baseline scans were performed 24 +/- 2 h apart in all patients before initiation of osimertinib treatment (Figure 3A). A modest, but significant increase in mean ^18^F-FDG normalized standard uptake value (nSUV) was observed in the enhancing tumor referenced to non-tumor-bearing cerebellum^41^ between the first and second pretreatment baseline scans (Figure 3B–C). Double baseline ^18^F-FDG nSUV mean was 0.97 with an overall increasing trend of the mean nSUV from first to second baseline scan (Figure 3D). Using a mixed-effects regression model, we estimated the percent change in mean ^18^F-FDG nSUV between the 2 baselines around 3.07% (Figure 3E; 95% CI 0.7%–5.5%; *P* = .009). Generally, repeated baseline evaluations confirmed a high repeatability of ^18^F-FDG PET measurements, with a consistent increase in nSUV over time between the 2 baseline scans, presumably due to the growing tumor.

### Osimertinib Shows a Minimal, but Quantifiable ^18^F-FDG PET Imaging Response in Human Recurrent GBM

**Figure 4.**
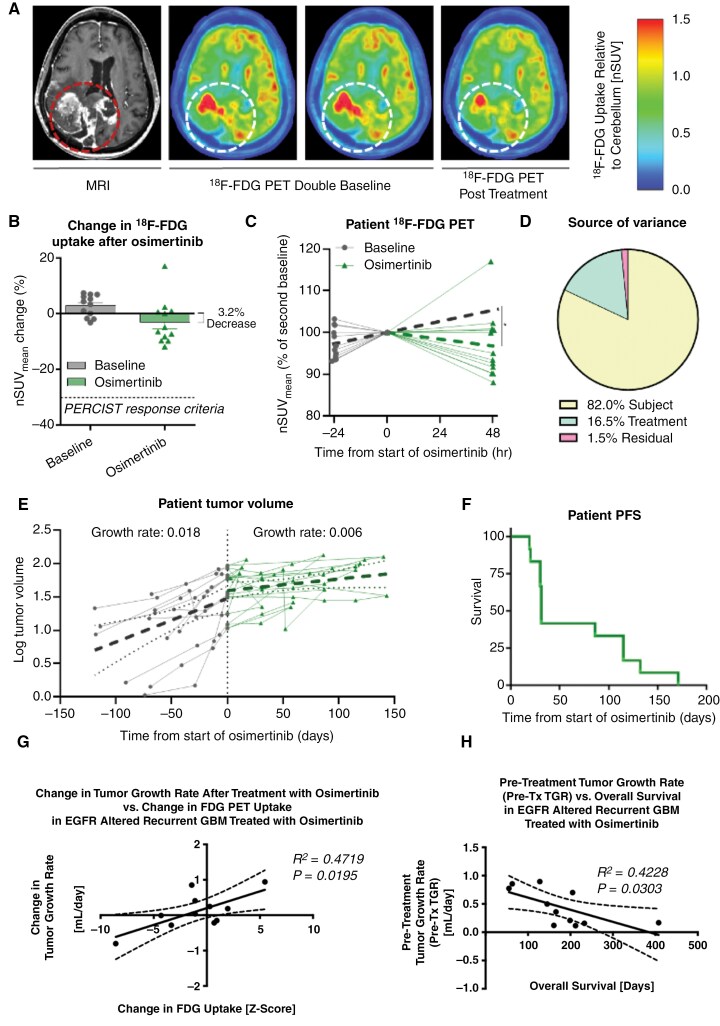
**Clinical** ^**18**^ **F-FDG PET imaging and anatomic MRI response to osimertinib.** (A) Example anatomical MRI, double baseline ^18^F-FDG PET scans, and response to osimertinib ^18^F-FDG PET scans in a single GBM patient. (B) Change in ^18^F-FDG uptake after osimertib treatment for each patient. No patients met the PERCIST response criteria (dotted line). (C) Individual patient nSUV_mean_ trajectories of their double baseline and treatment response. Projected linear rate of changes are plotted (dotted line) and a significant decrease in the rate of change after osimertinib treatment is observed. (D) An analysis of variance details the sources of variance with subject variability being the largest contributor to heterogeneity in ^18^F-FDG PET measurements. (E) Log of contrast enhancing tumor volume over time for each patient before and after osimertinib treatment. Using a mixed-effects regression model, linear growth rates are plotted (dotted lines) before and after start of osimertinib treatment. (F) Kaplan–Meier curve of PFS.

Upon completion of 2 pre-treatment ^18^F-FDG PET scans and a pre-treatment MRI exam, patients were treated with 240 mg osimertinib q.d. for 3 doses followed by a ^18^F-FDG PET scan 48 h after treatment administration to evaluate the osimertinib-induced attenuation in tumor glucose uptake. Some (4/12) patients displayed a visible and quantifiable decrease in ^18^F-FDG uptake within the contrast enhancing regions of the tumor relative to their double baseline scans after treatment (**Figure 4A**). The greatest change was a −12% decrease in ^18^F-FDG uptake (**Table 2**), and there was no statistically significant decrease in uptake relative to the second baseline scan when considering the entire patient cohort (*P* = .329). Thus, no patients had a metabolic response (> 30% reduction in SUV) according to the Positron Emission Tomography (PET) Response Criteria (PERCIST) (**Figure 4B**).^37^ There was no detectable correlation between patient glucose levels and ^18^F-FDG uptake (**Supplementary Figure S3**). When the within-subject variability was accounted for using the 2 pre-treatment baseline scans, 4 of the 12 patients (~33%) showed a statistically significant decrease in ^18^F-FDG uptake within the contrast enhancing tumor, 2 of 12 (~17%) showed a significant increase in uptake, and 6 of 12 (50%) did not exhibit a significant post-treatment change in ^18^F-FDG uptake (**Table 2**).

**Table 2.** Radiographic, Metabolic, and Clinical Response.

Subject ID	EGFR Status	PTEN Status^***^	Time on Treatment [Days]	mRANO PFS [Days]	Progressed on Drug? (vs. Censored)	mRANO Best Response	Change in ^18^F-FDG PET (Immediate Pre vs. Post) [%ΔnSUV/2 days]	PERCIST Best Response	Change in ^18^F-FDG PET Uptake in Enhancing Tumor [Z-Score**]	Significant Change in ^18^F-FDG PET Measurements [Z-Score > 1.645]^**^	Overall Survival [Days]
1	Amp	Mutant (no FoM)	121	115	Yes	SD	−8.3	SD	−1.11	0	233
2	Amp, EGFRvIII; R252C	Intact	115	115	Yes	SD	−9.8	SD	−8.57	↓	128
3	Amp, G598V	Mutant	133	132	Yes	SD	0.0	SD	0.70	0	205
4	Amp, EGFR exon20 insertion (H773_V774insAH); EGFRvIII; V774M	Loss	18	19	Yes	PD	17.0	SD	5.47	↑	55
5	Amp, EGFRvIII; EGFRvIVa; R222C	Intact	87	31	Yes	PD	−9.8	SD	−1.42	0	166
6	Amp, EGFRvIII; A289T	Loss	171	171	Yes	SD	0.5	SD	0.94	0	408
7	Amp, EGFRvIII; C291X	Intact	30	31	Yes	PD	−12.0	SD	−3.34	↓	63
8	Amp, EGFRvIII; T263P	Intact	20	20	No^*^	N/A^*^	−6.6	SD	−93.02	↓	103
9	Amp, EGFRvIII	Intact	30	30	No	SD	−5.0	SD	−4.31	↓	199
10	Amp, EGFRvIII	Intact	86	86	Yes	SD	−7.4	SD	−1.10	0	145
11	Amp	Intact	54	30	Yes	PD	2.2	SD	0.28	0	212
12	Amp	Intact	59	31	Yes	PD	0.8	SD	1.97	↑	161

^*^Withdrew from study.

^**^Relative to patient-specific variability measured with double baseline PET scans.

^***^PTEN status determined by Foundation Medicine or FISH. See **Supplementary Table S2**. Amp = Amplified.

While we did not observe a significant decrease in mean ^18^F-FDG nSUV levels 48 h after treatment relative to the most contemporary pre-treatment PET scan, we did observe a modest albeit significant decrease in the *linear rate of change* in ^18^F-FDG nSUV within the enhancing tumor following treatment, if the pre-treatment changes were taken into consideration (**Figure 4C**). If we extrapolate the positive rate of change in ^18^F-FDG nSUV observed before treatment using the 2 baseline PET scans, we estimate a significant decrease in the expected ^18^F-FDG nSUV growth rate averaging −4.5% per day (*P* = .01) with 95% C.I. (−1.1%, −7.9%). Results of an analysis of variance suggests the largest contributor to heterogeneity in ^18^F-FDG PET measurements was subject variability, which accounted for 82% of the total variance (**Figure 4D**), while treatment effects explain only 16.5% of the total variance. This finding is compatible with our understanding of inherent heterogeneity in GBM and across patient-level clinical characteristics. Additionally, these results suggest osimertinib induces a small change in glucose metabolism in some *patients with* recurrent GBM. Overall, a small but statistically significant decrease in the rate of change was observed with osimertinib treatment.

To determine whether osimertinib treatment influenced bulk tumor growth kinetics, the volume of contrast enhancing tumor was measured on 2–4 MRI scans before treatment, a pre-treatment baseline MRI scan within days of the first dose of osimertinib, and after treatment every ~8 weeks until osimertinib treatment was stopped or disease progression occurred (**Figure 4E**). Using a mixed effects regression model, we estimated a daily linear growth rate before treatment using *log*-transformed tumor volume (cm^3^) to be 0.018 (95% CI [0.013, 0.022]; *P* < .001). After treatment, the average growth rate changed by −1.2% per day (95% CI [−0.005, −0.019]; *P* < .001). There were no radiographic responses and no patients achieved 6-month PFS according to mRANO. Median PFS was 31 days (**Figure 4F**), while median OS was 164 days and no difference in OS was observed in patients exhibiting a best mRANO response of stable versus progressive disease (**Table 2**; *P* = .1789). It is important to note that the effects of changes in corticosteroid use were not considered in the model but were considered when evaluating mRANO response.

While there was no evidence of clinical benefit, the change in contrast enhancing tumor growth rate after treatment was significantly correlated with change in ^18^F-FDG uptake (**Figure 4G**; *R*^*2*^* = *0.4719, *P = .0195*) and pre-treatment contrast enhancing tumor growth rate was inversely correlated with overall survival (**Figure 4H**; *R*^2^ = 0.4228, *P =* .0303). Together, these data support the hypothesis that ^18^F-FDG PET is a predictive pharmacodynamic marker of EGFR inhibition and osimertinib does not sufficiently inhibit EGFR in recurrent GBM.

## Discussion

Aberrant EGFR signaling drives GBM glycolysis,^2,19^ raising the potential for ^18^F-FDG PET to serve as a non-invasive imaging surrogate for EGFR TKI tumor pharmacodynamics and as a predictive response biomarker in GBM patients.^22^ Osimertinib is a clinically approved drug for the treatment of EGFR-mutant NSCLC, due to the ability of it to inhibit the EGFR kinase found in these tumors. Although our study was not designed to statistically evaluate the effect of osimertinib across the spectrum of EGFR altered glioblastoma, these data showed that osimertinib failed to robustly inhibit ^18^F-FDG uptake in this cohort of EGFR-altered GBM patients per the PERCIST criteria. The minimal effect observed for osimertinib on tumor ^18^F-FDG consumption in these tumors was linked to only marginal antitumor clinical activity. Concurrent preclinical studies confirmed the limited impact on ^18^F-FDG uptake in EGFR-altered GBM tumor, suggesting this limitation may be due to inability of the EGFR TKI to robustly inhibit extracellular domain mutant EGFR signaling in GBM. This is in contrast with its significant impact on EGFR signaling, ^18^F-FDG uptake, and anti-tumor responses in an intracranial model of EGFR kinase mutant NSCLC, its approved clinical indication. The modest changes in FDG uptake observed both preclinically and clinically align with osimertinib’s limited ability to fully inhibit GBM-specific EGFR alterations, suggesting that more potent inhibition may be necessary to achieve clinically meaningful metabolic and therapeutic effects.

As a surrogate of tumor burden for clinical management, amino acid PET is preferred over ^18^F-FDG PET because ^18^F-FDG PET has elevated background signal from the normally high glucose metabolism in the brain.^45–47^ However, we theorized ^18^F-FDG PET may be useful, not as a measure of tumor burden as is used in a broad range of cancers,^48–50^ but as a pharmacodynamic imaging biomarker for quantifying changes in glucose metabolism. Our data suggests that, if pre-treatment changes in ^18^F-FDG uptake are taken into consideration, a small but significant decrease in the rate of change in ^18^F-FDG uptake in areas of enhancing tumor after administration of osimertinib can be observed. Importantly, our data shows little measurement variability that could not be accounted for by patient heterogeneity and treatment response, supporting the use ^18^F-FDG PET as a clinically reliable imaging biomarker for EGFR inhibition in human GBM, which could negate the need for repeat biopsies to confirm pharmacodynamic changes. This potential non-invasive way to monitor glucose metabolism in response to therapy would be particularly important for GBM EGFR inhibition, as tumor tissue-based studies have shown a high degree of heterogeneity in pharmacodynamic response^15^ and drug exposure in GBM.^16,51^

In addition to demonstrating the use of molecular imaging as a biomarker for pharmacodynamic changes and antitumor activity, our study illustrated how preclinical GBM models can be used concurrently to confirm clinical observations and learn from a failed trial. By testing preclinical models in parallel with a clinical trial, we confirmed that osimertinib demonstrates disparate results in a GBM compared to NSCLC brain metastasis and this is likely due to multiple factors. For example, although brain penetrant, the available free drug exposures of osimertinib in the mouse brain are approximately 20–40% of that to plasma^25–27^ (**Supplementary Figure S4**). Given that osimertinib demonstrates reduced activity against EGFR alterations specific to GBM, particularly extracellular domain mutations and amplifications, compared to the kinase domain mutations found in NSCLC^52^ (**Supplementary Figure S5**), our results suggest that osimertinib can achieve sufficient exposures to inhibit kinase domain mutant *EGFR*, like those observed in NSCLC *EGFR*; however, these free drug exposures appear inadequate to robustly inhibit the *EGFR* alterations found in GBM. Notably, patients harboring EGFR extracellular domain mutations (R252C, G598V, and A289T) showed numerically longer PFS, consistent with previous work suggesting potential sensitivity of these mutations to EGFR inhibition. However, the small sample size precludes definitive conclusions about predictive biomarkers. The observed increase in EGFR activity and downstream signaling in some GBM samples may reflect compensatory pathway activation or feedback mechanisms that are specific to GBM biology. This unexpected finding further highlights the complexity of targeting EGFR in GBM and suggests that different therapeutic approaches may be needed in GBM compared to NSCLC. These findings suggest multiple factors should be considered (eg, free drug exposure, activity against GBM-specific *EGFR* alterations) for an EGFR TKI to be a promising drug candidate for *EGFR*-mutant GBM. As new EGFR TKIs are emerging that report both high unbound CNS exposure and activity against GBM-specific EGFR alterations (eg, ERAS801 (NCT05222802), JCN037,^52^ BDTX-1535,^53^ WSD0922-FU^54^), future FDG-based imaging studies using these clinical compounds could be warranted.

## Conclusion

The current study demonstrated the ability of non-invasive metabolic imaging to evaluate promising EGFR-inhibitor candidates both in the preclinical and clinical settings under similar therapeutic exposures. Results for concurrent preclinical and clinical data confirmed osimertinib’s limited impact on GBM.

## Supplementary Material

vdaf022_suppl_Supplementary_Figures_S1-S5_Tables_S1-S2

## Data Availability

The deidentified data that support the findings of this study are privately owned and will be made available upon reasonable request, in accordance with *Neuro-Oncology Advances’* data sharing policy. Interested researchers may contact the corresponding author to request access to the underlying data used in this study, subject to institutional review process and data use agreements.
