# Correction to: A comparative study of preclinical and clinical molecular imaging response to EGFR inhibition using osimertinib in glioblastoma

**DOI:** 10.1093/noajnl/vdaf119

**Published:** 2025-06-25

This is a correction to: Benjamin M Ellingson, Quincy Okobi, Robert Chong, Rhea Plawat, Eva Zhao, Andrei Gafita, Ida Sonni, Saewon Chun, Emese Filka, Jingwen Yao, Donatello Telesca, Shanpeng Li, Gang Li, Albert Lai, Phioanh Nghiemphu, Johannes Czernin, David A Nathanson, Timothy F Cloughesy, A comparative study of preclinical and clinical molecular imaging response to EGFR inhibition using osimertinib in glioblastoma, *Neuro-Oncology Advances*, Volume 7, Issue 1, January-December 2025, vdaf022, https://doi.org/10.1093/noajnl/vdaf022

In the originally published version of this manuscript, the author Quincy Okobi’s name was incorrectly given as Qunicy Okobi. This error has been corrected.

