# Supplementary material for: A comparative study of preclinical and clinical molecular imaging response to EGFR inhibition using osimertinib in glioblastoma
Source: Neurooncol Adv. 2025 Feb 19;7(1):vdaf022. doi: 10.1093/noajnl/vdaf022 (PMC11883343; doi:10.1093/noajnl/vdaf022)
Supplement: vdaf022_suppl_Supplementary_Figures_S1-S5_Tables_S1-S2 [file vdaf022_suppl_supplementary_figures_s1-s5_tables_s1-s2.docx]

**Supplementary Materials:**

**Supplemental Table S1: Treatment Emergent Adverse Events and Toxicities.**

| **TEAE Description** | **Grade 3** | **Grade 4** |
| --- | --- | --- |
| Investigations- AST elevation | 1 |  |
| Investigations- ALT elevation | 1 |  |
| Pulmonary embolism/Serious Adverse Event (SAE) | 1 |  |
| Respiratory- infection- pneumonia | 1 |  |
| Diarrhea with C diff/SAE | 1 |  |
| Vomiting | 1 |  |
| Procedural injury- Wound dehiscence/SAE | 1 |  |
| Nervous System Disorders- Headache | 1 |  |
| Hydrocephalus/SAE | 1 |  |
| Nervous System- Encephalopathy/SAE | 1 |  |
| Seizure/SAE | 1 |  |
| Nervous System Disorders- Somnolence | 1 |  |
| Intracranial hemorrhage SAE | 1 |  |
| Vascular- Deep Vein Thrombosis, (DVT)/SAE | 1 |  |
| Metabolism- Hyponatremia | 2 |  |
| Vasogenic/Cerebral Edema (MRI) | 2 |  |
| Mental status changes/SAE | 4 |  |

**Supplemental Table S2: PTEN Status**

| Subject ID | **Consensus PTEN Status** | **PTEN status (FISH)** | **PTEN status (FoM seq)** | **PTEN status (detailed)** |
| --- | --- | --- | --- | --- |
| 1 | **Mutant (no FoM)** | loss (UCLA FISH) | *no FoM performed* | FISH (UCLA) indicates positive for monosomy 10 (PTEN loss), no FOM performed; WEX indicates (1) copy loss & C136Y mutation |
| 2 | **Intact** | *BTTR does not have path report* | no alteration detected | No info on UCLA FISH; FoM performed, no PTEN alterations detected; no WEX, not processed by lab |
| 3 | **Mutant** | loss (UCLA FISH) | mutation (R130*) | FISH (UCLA) indicates positive for monosomy 10 (PTEN loss), FoM indicates PTEN R130*; WEX indicates (1) copy loss & R130* mutation |
| 4 | **Loss** | Monosomy (UCLA FISH) | loss | FISH (UCLA) indicates positive for monosomy 10 (PTEN loss), FoM indicates PTEN loss; WEX indicates (2) copy loss/deep deletion & no mutation |
| 5 | **Intact** | *FISH for PTEN not performed* | no alteration detected | No info on UCLA FISH; FoM performed, no PTEN alterations detected; WEX indicates (1) copy loss & G129E mutation |
| 6 | **Loss** | *FISH for PTEN not performed* | loss | No info on UCLA FISH; FoM indicates PTEN loss; no WEX data available |
| 7 | **Intact** | *FISH for PTEN not performed* | no alteration detected | No info on UCLA FISH; FoM performed, no PTEN alterations detected; no WEX data available |
| 8 | **Intact** | *FISH for PTEN not performed* | no alteration detected | No info on UCLA FISH; FoM performed, no PTEN alterations detected; WEX indicates (1) copy loss & no mutation detected |
| 9 | **Intact** | *BTTR does not have path report* | no alteration detected | No info on UCLA FISH; FoM performed, no PTEN alterations detected; no WEX, not processed by lab |
| 10 | **Intact** | loss (UCLA FISH) | no alteration detected | FISH (UCLA) indicates deletion 10q (PTEN loss), FoM performed, no PTEN alterations detected; no WEX data available |
| 11 | **Intact** | loss (UCLA FISH) | no alteration detected | FISH (UCLA) indicates deletion 10q (PTEN loss), FoM performed, no PTEN alterations detected; WEX indicates (1) copy loss & no mutation detected |
| 12 | **Intact** | Intact | no alteration detected | FISH (UCLA) indicates Monosomy 10 (PTEN loss), FoM performed, no PTEN alterations detected; WEX indicates (1) copy loss & no mutation detected |

FoM = Foundation Medicine

**Fig. 1. Clinical determination of ^18^F-FDG PET region of interest.** All FDG PET images (A) were linearly registered (6 degrees of freedom) to (B) pre-treatment, post-contrast T1-weighted MRI scans, resulting in (C) fusion of the FDG PET and the MRI images. Using this alignment, contrast enhancing regions of interest (D) were contoured on post-contrast T1-weighted images and directly applied to the respective FDG PET images for FDG uptake measurement (E).

**
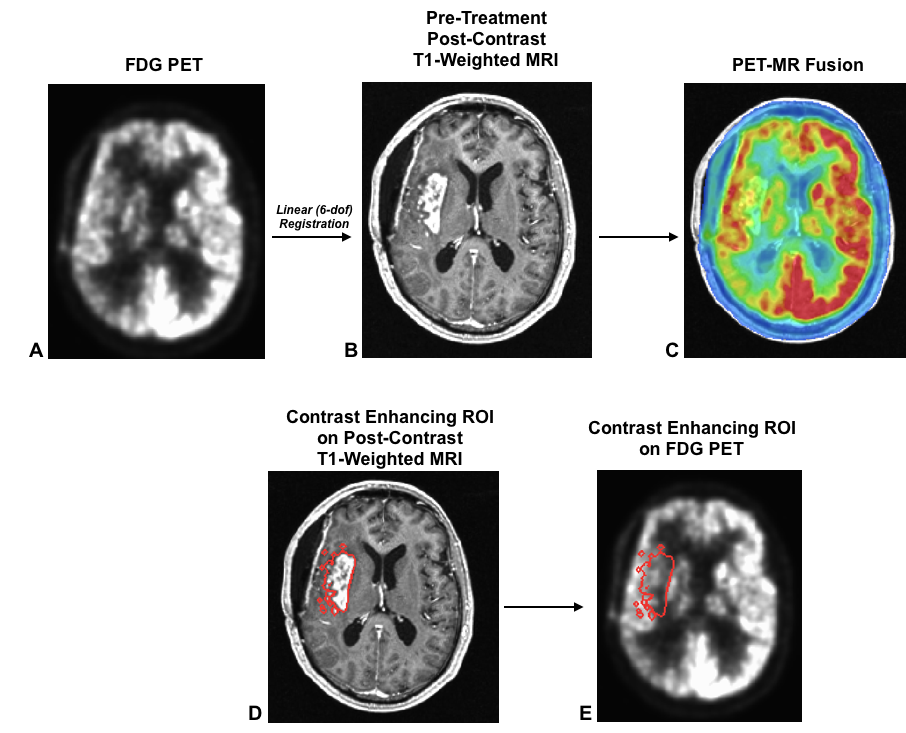
**

**Supplementary Fig. S2: Osimertinib maximum tolerated dose**. Treatment regimens of 25 mg/kg q.d., 25 mg/kg b.i.d., and 33 mg/kg q.d. were evaluated in Female NSG mice (n=5).

**Supplementary Fig. S3: Osimertinib patient glucose levels and nSUV_mean_**. (A) Patient glucose levels at the time of ^18^F-FDG PET imaging compared against the measured tumor nSUV_mean­­_. (B) Patient glucose levels and nSUV_mean_ were normalized and plotted and show no correlation between patient glucose and nSUV_mean_.

**
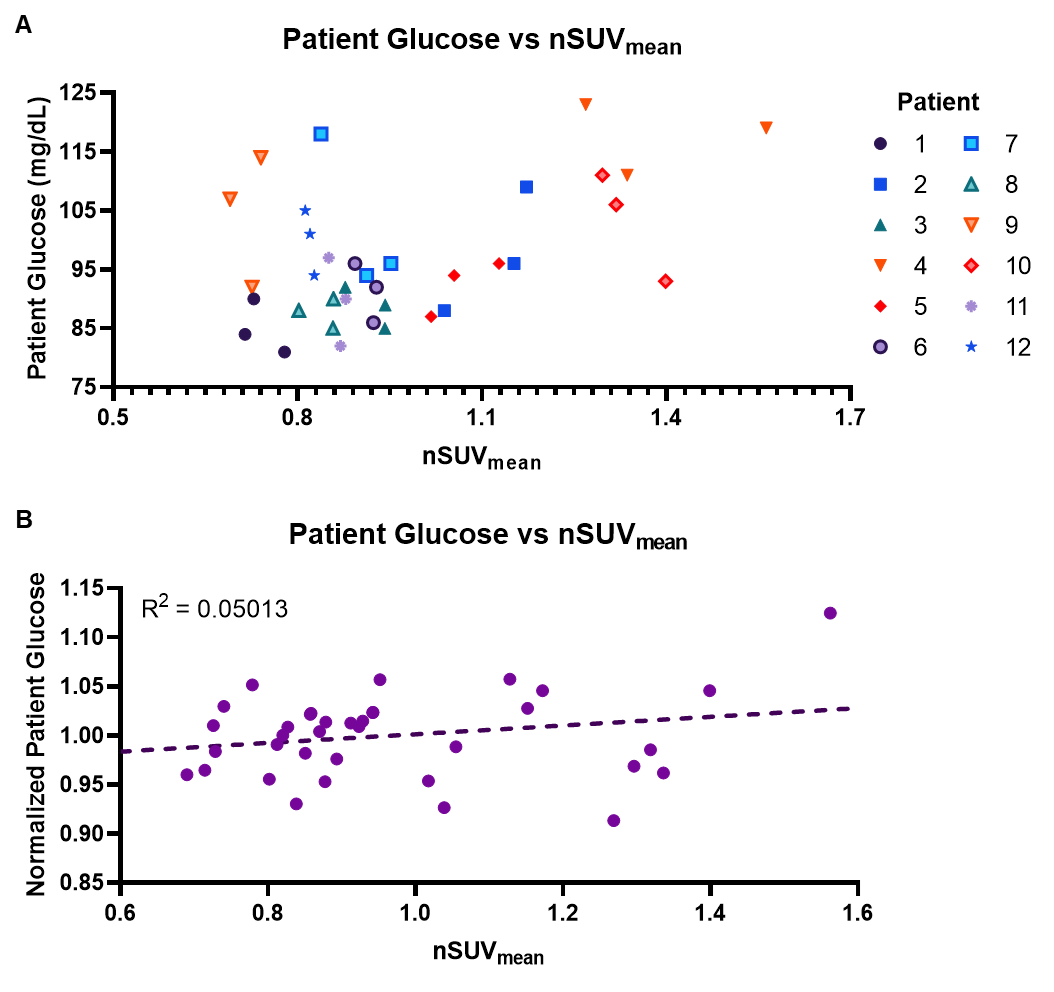
**

**Supplementary Fig. S4: Unbound plasma and brain concentrations of osimertinib at 25 mg/kg.** CD-1 mice were treated over a 24-hour period and unbound, free drug concentrations were measured in plasma (A) and brain (B) tissue at the specified timepoints (n=2 mice per time point). Average concentrations in each tissue type noted with the dotted line. (C) Pharmacokinetic parameters of osimertinib in mice. Fraction unbound in mouse plasma (Fu plasma) and mouse brain homogenate (Fu brain) were determined using RED dialysis plates for each EGFR TKI to calculate Kp,uu.

**Supplementary Fig. S5: Biochemical activity of osimertinib in EGFR ex19 del NSCLC.** (A) Immunoblots of *in vitro* PC9 NSCLC cells treated with the specified doses of osimertinib. (B) Quantification of (A) plotted against pEGFRvIII and pEGFR WT activity of osimertinib in Tsang *et al.^51^*

**SUPPLEMENTAL MATERIAL REFERENCES**

**1.** Chung E, Yamashita H, Au P, Tannous BA, Fukumura D, Jain RK. Secreted Gaussia luciferase as a biomarker for monitoring tumor progression and treatment response of systemic metastases. *PLoS One.* Dec 15 2009;4(12):e8316.

**2.** Nathanson DA, Armijo AL, Tom M, et al. Co-targeting of convergent nucleotide biosynthetic pathways for leukemia eradication. *J Exp Med.* Mar 10 2014;211(3):473-486.

**3.** Dai H, Marbach P, Lemaire M, Hayes M, Elmquist WF. Distribution of STI-571 to the brain is limited by P-glycoprotein-mediated efflux. *J Pharmacol Exp Ther.* Mar 2003;304(3):1085-1092.
